# Publicly available online activity restrictions after reverse total shoulder arthroplasty demonstrate marked variability

**DOI:** 10.1016/j.jsea.2026.100076

**Published:** 2026-08-11

**Authors:** Kyle K. Obana, Joshua D. Winward, Andrew J. Luzzi, Michael L. Knudsen, Charles M. Jobin, William N. Levine

**Affiliations:** a Department of Orthopedic Surgery, NewYork-Presbyterian Hospital, Columbia University Irving Medical Center, New York, NY, USA; b New York Medical College, Valhalla, NY, USA; c Investigation Performed at Columbia University Irving Medical Center, New York, NY, USA

**Keywords:** Rehabilitation, Protocols, Restrictions, Weight, Post-operative, Guidelines, Limitations, Lifetime

## Abstract

**Background:**

Physicians may implement post-operative restrictions following reverse total shoulder arthroplasty (rTSA) due to concerns of post-operative instability and accelerated polyethylene wear. However, no data support the amount of weight patients should limit above and below horizontal. The current study analyzes available online provider, group practice, and hospital protocols pertaining to restrictions following rTSA. The authors hypothesize that protocols will have substantial variability in both rehab milestones and lifetime lifting limits.

**Methods:**

The search query “reverse total shoulder arthroplasty weight restrictions” was entered on January 25, 2026. The first 50 websites that met inclusion criteria were included. Websites were evaluated for presence of lifetime weight restrictions, weight restriction above and below horizontal, duration of immobilization, timing of physical therapy, timing of range of motion (ROM), timing of strengthening exercises, timing of return-to-sport (RTS), and incorporation of scientific references.

**Results:**

Website categories were 23 (46%) single provider, 15 (30%) group practice, and 12 (24%) hospital-based. Mean post-operative immobilization was 4.7 ± 1.4 weeks (2 to 8 weeks), with 79.1% recommending ≥4 weeks of immobilization. Shoulder passive ROM was initiated at 0.9 ± 1.2 weeks, active-assisted ROM at 4.2 ± 1.9 weeks, and active ROM at 5.8 ± 1.9 weeks. Five (10%) websites specified no lifetime weight restrictions, 20 (40%) implemented lifetime restrictions, and 25 (50%) did not specify. Maximum weight below horizontal was 11.8 ± 4.1 kgs (26.0 ± 9.1 lbs) and above horizontal was 9.3 ± 4.0 kgs (20.4 ± 8.9 lbs). Twenty-three (46%) websites mentioned RTSs. Fifteen of these websites (68.2%) reported RTSs at 3.6 ± 0.6 months. Twelve websites (24%) mentioned golf, 10 (20%) racquet sports, and 7 (14%) swimming.

**Conclusion:**

Online protocols are highly variable with respect to lifetime weight restrictions, post-operative rehabilitation, and RTS expectations following rTSA. Despite no formal recommendations in the literature regarding lifetime weight restrictions, 40% of websites specified weight restrictions below and above horizontal. Future studies should elucidate safe weight limit thresholds and specific therapy protocols to guide formal recommendations.

Reverse total shoulder arthroplasty (rTSA) is a widely and increasingly performed surgery to treat a variety of glenohumeral pathologies. Compared to the native glenohumeral joint, rTSA reverses the convexity and concavity of the articulating surfaces to address the biomechanical challenges posed by a deficient rotator cuff, which anatomic total shoulder arthroplasty (aTSA) does not adequately resolve.^6^^,^^29^ This reversed design of a concave humeral tray and convex glenosphere medializes and distalizes the center of rotation (COR).^19^^,^^29^ This non-anatomic COR increases the resting deltoid tension and the deltoid moment arm, thereby improving the efficiency of the deltoid to forward elevate the shoulder and increasing stability through compressive forces. Although rTSA is advantageous at allowing the deltoid to compensate for a deficient rotator cuff, many surgeons implement post-operative and lifetime restrictions to avoid instability, stress at the bone-implant interface, and accelerated implant wear.

Patients living with rTSA must be able to use the extremity to perform functional tasks related to occupational demands and activities of daily living, such as carrying items, lifting objects from the ground, and placing items overhead. Patients requiring crutches, walkers, or wheelchairs must use their upper extremities to offload weight from their lower extremities.^20^ The ability to engage in sports and other forms of physical activity is also beneficial for quality of life and overall health and should be maintained. Some surgeons may impose lifetime weight restrictions both above and below shoulder level due to concerns regarding stress at the glenohumeral interface, accelerated implant wear, and subsequent instability.^1^^,^^4^ However, there is currently no consensus regarding permanent weight restrictions, timing of immobilization, or initiation of certain movements following rTSA.^4^^,^^8^^,^^13^^,^^16^^,^^28^ For patients who are given restrictions, there are no data to support the weight patients should limit above and below horizontal. The current study characterizes immobilization duration, rehabilitation milestone timing, return-to-sport (RTS) guidance, and lifetime lifting limits in the first 50 patient-facing online protocols. The authors hypothesize that most protocols will have substantial variability in both rehabilitation milestones and lifetime lifting limits.

## Materials and methods

### Inclusion criteria

This study did not require institutional review board approval. The Google search was conducted out of New York, New York, on January 25, 2026. Using a clean Google Chrome browser with cleared cache/cookies, logged out of profiles, and in incognito mode, the search query “reverse total shoulder arthroplasty weight restrictions” was entered. Inclusion criteria were single provider, group practice, or hospital websites that pertained to rTSA. Exclusion criteria were sponsored websites, general medical websites not affiliated with a health-care organization (eg, WebMD, Healthline), videos, scientific research articles, blogs, social media posts, non-rTSA protocols (ie, aTSA, hemiarthroplasty), and the inability to differentiate between anatomic and rTSA recommendations/guidelines.

### Data collection

Two authors (J.D.W., K.K.O.) performed data collection separately for all websites. Data collection discrepancies between the 2 authors were resolved by a third author (A.J.L.). Websites were evaluated for post-operative protocols and lifetime restrictions including lifetime weightlifting restrictions, duration of post-operative immobilization, timing of initiation of physical therapy (PT), timing of passive range of motion (PROM), active-assisted range of motion (AAROM), and active range of motion (AROM), timing of shoulder strengthening exercises, and mention of RTS. Mention of lifetime weight restrictions were categorized as yes, no, or does not specify. If lifetime weight restrictions were recommended, then the amount of weight allowed (kgs) below and above horizontal were recorded. Single providers were recorded as being shoulder and elbow fellowship-trained versus other. Websites were evaluated for inclusion of 1 or more scientific references.

### Definitions

Lifetime weight restriction was defined as any permanent, indefinite, or “for life” restriction that was to remain after completion of post-operative rehabilitation. Weight restrictions above horizontal were defined as restrictions above 90° shoulder abduction, above shoulder height, above shoulder level, or overhead. Post-operative immobilization was defined as the requirement to wear a sling in the house, public, or while sleeping. All websites with missing biographies and/or ambiguous fellowship training (eg, sports medicine and shoulder reconstruction) had Doximity, hospital profiles, and/or publicly available CVs evaluated to identify whether the surgeon pursued a shoulder and elbow fellowship. This was performed after all websites were included to avoid inducing further bias.

### Statistical analysis

Descriptive statistics are used. Continuous variables are reported as mean ± standard deviation with range. Categorical variables are reported as frequency (percentage). Intraclass correlation coefficients were calculated to determine the reliability between the 2 authors. Reliability is defined as <0.0 = poor, 0.00 to 0.20 = slight, 0.21 to 0.40 = fair, 0.41 to 0.60 = moderate, 0.61 to 0.80 = substantial, and 0.81 to 1.00 = almost perfect.^18^ All statistical analyses were performed using STATA/MP software (version 13.0; StataCorp LLC, College Station, TX, USA).

## Results

### Demographics

**Figure 1:**
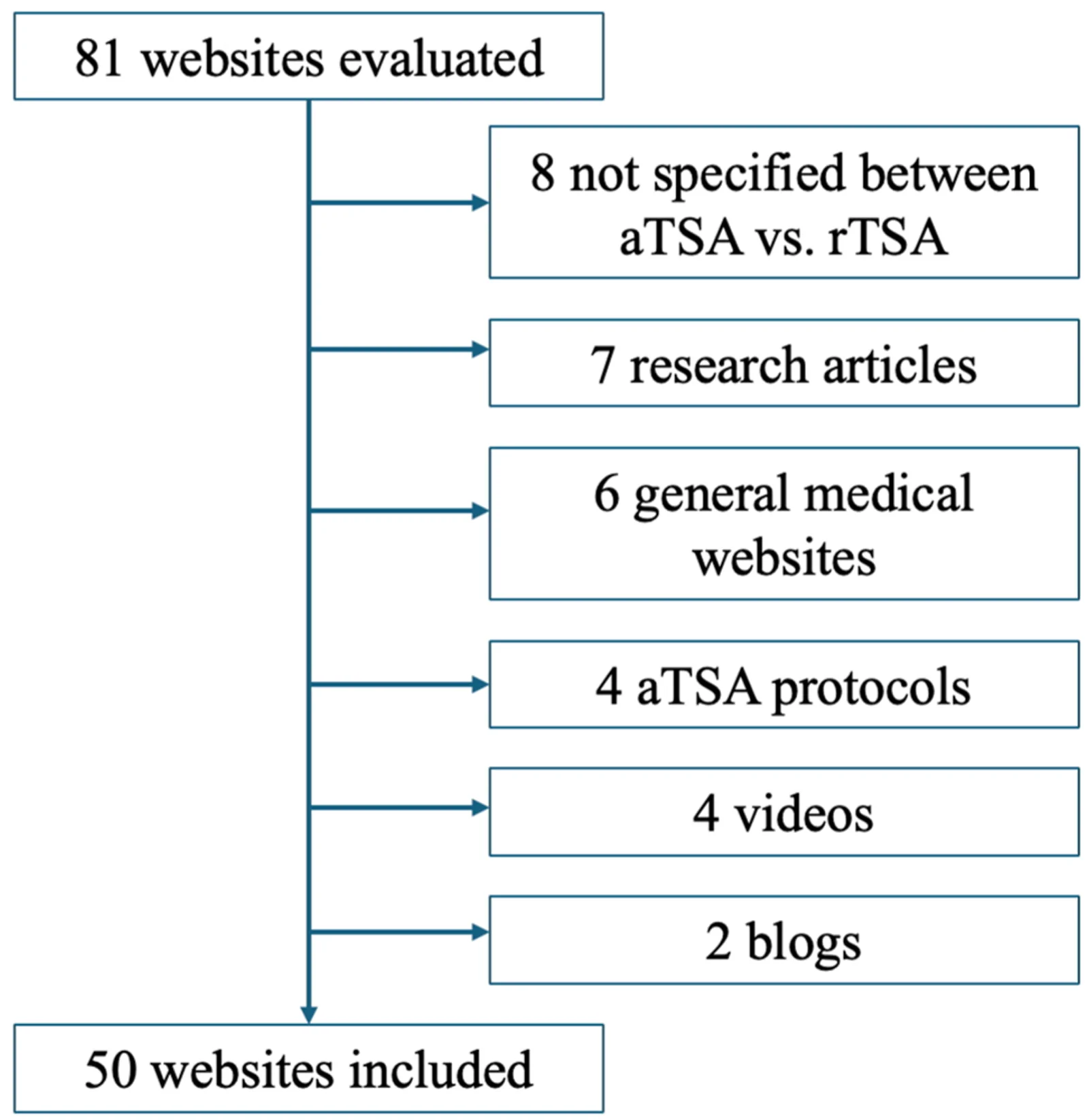
Flowchart for included and excluded websites based on the initial query. *aTSA*, anatomic total shoulder arthroplasty; *rTSA*, reverse total shoulder arthroplasty.

Eighty-one websites were evaluated and the first 50 that met inclusion criteria were included in accordance with prior studies (Fig. 1).^2^^,^^3^ The website categories were 23 (46%) single provider, 15 (30%) group practice, and 12 (24%) hospital. Fourteen (60.9%) of the single providers completed a sports medicine fellowship, 7 (30.4%) completed a shoulder/elbow fellowship, and 2 (8.7%) completed a hand/upper extremity fellowship.

### Post-operative rehabilitation

Duration of post-operative immobilization was 4.7 ± 1.4 weeks (2 to 8 weeks) (Table I). Forty-three (96.0%) specified duration of post-operative immobilization. Out of the protocols that specified duration of immobilization, 79.1% recommended ≥4 weeks of immobilization (Table II). Shoulder PROM is initiated at 0.9 ± 1.2 weeks post-operatively (0 to 6 weeks), AAROM at 4.2 ± 1.9 weeks (0 to 6 weeks), and AROM at 5.8 ± 1.9 weeks (0 to 12 weeks). Periscapular strengthening begins at 8.9 ± 2.8 weeks (4 to 12 weeks).

**Table I:** Timing of post-operative rehabilitation milestones and maximum weight restrictions above and below horizontal, as well as the proportion of protocols that specified these metrics.

Movement/restriction	Mean ± STD	N (%)
Immobilization	4.7 ± 1.4 weeks	42 (84%)
PROM	0.9 ± 1.2 weeks	36 (72%)
AAROM	4.2 ± 1.9 weeks	35 (70%)
AROM	5.8 ± 1.9 weeks	36 (72%)
Strengthening	8.9 ± 2.9 weeks	37 (74%)
Maximum weight below horizontal	11.8 ± 4.1 kgs (26.0 ± 9.1 lbs)	20 (40%)
Maximum weight above horizontal	9.3 ± 4.0 kgs (20.4 ± 8.9 lbs)	20 (40%)

*PROM*, passive range of motion; *AAROM*, active-assisted range of motion; *AROM*, active range of motion; *STD*, standard deviation.

**Table II:** Proportion of websites specifying duration of post-operative immobilization.

Immobilization (weeks)	N (%)
2	3 (7.0%)
3	6 (14.0%)
4	14 (32.6%)
5	1 (2.3%)
6	18 (41.9%)
8	1 (2.3%)
Total	43 (100%)

### Restrictions and expectations

Five (10%) websites specified no lifetime weight restrictions, 20 (40%) implemented lifetime restrictions, and 25 (50%) did not specify. Fifteen (30%) websites specified a maximum weight below horizontal of 11.8 ± 4.1 kgs (6.8 to 22.7 kgs) (26.0 ± 9.1 lbs [15 to 50 lbs]) and 19 (38%) specified a maximum weight above horizontal of 9.3 ± 4.0 kgs (0 to 13.6 kgs) (20.4 ± 8.9 lbs [0 to 30 lbs]) (Table I). The most common maximum weight below horizontal was 11.3 kgs (25 lbs) (40%), while the most common maximum weight above horizontal was also 11.3 kgs (25 lbs) (42%) (Tables III and IV). Twenty-three (46%) websites mentioned RTSs, while the rest (54%) did not specify. Fifteen of these websites (68.2%) reported RTSs (eg, golf) at 3.6 ± 0.6 months (3 to 5 months). Twelve websites (24%) mentioned golf, 10 (20%) mentioned racquet sports (eg, tennis), and 7 (14%) mentioned swimming. Twelve websites (24%) included 1 or more scientific references.

**Table III:** Proportion of websites with specific below horizontal weight restrictions.

Below horizontal weight restrictions (kgs)	N (%)
6.8 (15 lbs)	2 (13.3%)
9.1 (20 lbs)	3 (20.0%)
11.3 (25 lbs)	6 (40.0%)
13.6 (30 lbs)	2 (13.3%)
18.1 (40 lbs)	1 (6.7%)
22.7 (50 lbs)	1 (6.7%)
Total	15 (100%)

**Table IV:** Proportion of websites with specific above horizontal weight restrictions.

Above horizontal weight restrictions (kgs)	N (%)
0	1 (5.3%)
1.4 (3 lbs)	1 (5.3%)
4.5 (10 lbs)	2 (10.5%)
6.8 (15 lbs)	1 (5.3%)
9.1 (20 lbs)	3 (15.8%)
11.3 (25 lbs)	8 (42.1%)
13.6 (30 lbs)	3 (15.8%)
Total	19 (100%)

### Intraclass correlation coefficients

Intra-class correlation coefficients demonstrated an almost perfect agreement for presence of lifetime weight restrictions (1.0), immobilization (0.96), PROM (0.89), AROM (0.85), AAROM (0.82), maximum weight below horizontal (0.82), maximum weight above horizontal (0.82), and timing of strengthening exercises (0.82).

## Discussion

The current study demonstrates that available online protocols for rTSA are highly variable with respect to permanent weightlifting restrictions and timing of rehabilitation milestones. Despite the paucity of studies justifying lifetime weight restrictions following rTSA, 40% of websites implemented specific weight limits below and above horizontal. The mean maximum weight limit below horizontal in this study was 11.8 kgs (range: 6.8 to 22.7 kgs) (26 lbs [range: 15 to 50 lbs]) while the mean limit above horizontal was 9.1 kgs (range: 0 to 13.6 kgs) (20 lbs [range: 0 to 30 lbs]). At the author's institution, there are no lifetime weight restrictions following rTSA and patients are advised to use their arm at their discretion. However, it is important to consider that surgery and implant-specific factors can increase implant wear, such as decreasing neck-shaft angle and reducing cup depth.^19^ Due to the altered biomechanics of rTSA relative to hemiarthroplasty or aTSA, many physicians implement post-operative and lifetime restrictions to avoid associated complications and accelerated implant wear. Future biomechanical studies may investigate weight thresholds at which implants become unstable.

Duration of post-operative immobilization in the current study was 4.7 weeks, with 79% implementing ≥4 weeks of immobilization. This is consistent with recommendations in the literature to protect the soft tissues.^7^ However, a recent prospective randomized controlled trial found no difference between 3 weeks of immobilization in an internal rotation sling versus no immobilization.^32^ At the author's institution, patients are immobilized in a sling with an abduction pillow for 2 weeks to allow for soft tissue rest. These patients should remain in the sling while sleeping and in public and are allowed to be out of it in safe environments (eg, home, office). Freehill et al^7^ found that surgeons in Europe primarily use simple slings following primary rTSA, regardless of subscapularis repair, while surgeons in the United States primarily use slings with an abduction pillow. Thus, there exists no superior option, at least as defined by the literature, regarding duration and form of post-operative immobilization following rTSA.

Currently, there is no superiority of home exercises versus formal PT and no consensus regarding timing of post-operative therapy following rTSA.^8^^,^^13^^,^^16^^,^^28^^,^^30^ A recent study surveying members of the American Shoulder and Elbow Surgeons (ASES) found 65% of providers allowed pendulums and 50% allowed PROM below shoulder level in first week.^11^ In the current study, post-operative shoulder range of motion (ROM) was initiated early, with PROM starting at 0.9 weeks post-operatively, AAROM at 4.2 weeks, and AROM at 5.8 weeks. Interestingly, initiation of PROM and AAROM began as early as 0 weeks in some protocols, and PROM as late as 6 weeks and AROM as late as 12 weeks in others. Variability in initiation of ROM following rTSA highlights the large variability in beliefs regarding timing of post-operative therapy. Recent randomized studies have demonstrated that early post-operative ROM may be beneficial and without increased risk of complications.^5^^,^^12^ However, the definition of “early ROM” varies with respect to muscle strengthening, planes of motions, and target angles allowed. As a growing body of literature emerges supporting early ROM following rTSA, so will a possible transition to accelerated rehabilitation protocols.

Ability to RTS is an important consideration post-operatively to improve cardiovascular fitness and health longevity. This study demonstrates that patients should expect to return to low-to-moderate demand sports within 4 months post-operatively based on provider recommendations. This is in concordance with the literature that demonstrates RTS at 3 to 6 months. Studies demonstrate RTS ranging from 40% to 100% for lower demand sports such as golf and swimming, with an overall rate of 77%.^10^^,^^14^^,^^22^^,^^26^^,^^27^^,^^31^^,^^33^ Only 14% of websites in the current study specified swimming as a sport patients may expect to return to post-operatively. This is likely due to concerns regarding unfavorable positioning of the implants during phases of certain strokes that may risk instability. However, Mousad et al^24^ surveyed 53 post-operative rTSA patients who were not given restrictions after 12 weeks post-operatively and found 100% could do freestyle, 49% could do breaststroke, 38% could do backstroke, and 15% could do butterfly. Mousad et al^24^ acknowledge that this may be attributed to lack of post-operative swimming restrictions after 12 weeks and intrinsic patient motivation to continue swimming.

A recent survey of surgeons in the ASES found that only 45% allow RTS following rTSA, although the authors were unable to elucidate the reasoning.^10^ Geyer et al^9^ analyzed RTS after rTSA and found that out of patients who gave up on sports/activities, 21.1% were fearful to return and 10.5% were discouraged to by their providers. Similarly, Tangtiphaiboontana et al^31^ found that fear of injury precluded 21% of patients from participating in sports post-operatively. Kim et al^17^ reported 37.5% of patients who did not RTS post-operatively were concerned about complications without having any discomfort. Setting favorable expectations may be beneficial for patients, as 43% of patients underwent rTSA in order to return to swimming.^24^ It is important to consider how biomechanical restrictions following rTSA (eg, limited internal rotation) may impede the ability to return to certain sports. In general, contact sports are not recommended following rTSA due to concerns for implant loosening and overload.^10^

### Implications for standardization

Currently, there are no evidence-based lifetime weight thresholds, and published protocols rarely cite supporting data. In the current study, only 24% of websites included 1 or more scientific references to support the post-operative protocols. This is an important factor, as surgeon's recommendations and patient expectations can influence post-operative activity and outcomes. Physical therapists must navigate the large variability between provider protocols, which can create an expectation mismatch, inconsistent counseling, and potential for non-adherence for patients. Development of a standardized rehabilitation protocol and lifetime restrictions following rTSA can help improve communication, post-operative expectations, shared decision-making, and patient outcomes.^15^^,^^21^^,^^23^^,^^25^

### Limitations

There are limitations to this study. Many protocols did not differentiate between home exercise programs and formal PT. This is likely attributed to similar effectiveness between both modalities to allow patients to begin ROM.^11^ Thus, timing of therapy was determined by any associated documentation. Second, these data are based off a limited, publicly available sample. Although this is not generalizable to all provider protocols, the authors used the first 50 available websites as a representative proxy for what patients may access online. It is possible that the exclusion of 31 websites may have induced bias in this study. However, the exclusion criteria were implemented to minimize inaccuracies associated with illegitimate or irreputable websites (eg, blogs, videos, social media, non–health-care organizations). Although these results may reflect recommendations by certified health-care individuals and organizations rather than all available online information, the objective of this study was to evaluate variability in published protocols, not all available online health-care information. Third, RTS timelines for each specific sport (eg, golf, swimming, tennis, lifting, running) were not possible due to lack of available information. To avoid variability in post-operative protocols, the authors suggest recommendations be established by the ASES regarding RTS timeline and post-operative restrictions following rTSA.

## Conclusion

Online protocols are highly variable with respect to lifetime weight restrictions, post-operative rehabilitation, and RTS expectations following rTSA. Despite no formal recommendations in the literature regarding lifetime weight restrictions, 40% of websites specified weight restrictions below and above horizontal. Additionally, protocols vary regarding ROM progression and muscle strengthening. Future studies should elucidate safe weight limit thresholds and specific therapy protocols to guide formal recommendations.

## Disclaimers:

Funding: No funding was disclosed by the authors.

Conflicts of interest: Michael L. Knudsen: MR Surgical Solutions, LLC: IP royalties and paid consultant. Charles M. Jobin: Acumed, LLC: Paid consultant; American Board of Orthopaedic Surgery, Inc.: board or committee member; American Shoulder and Elbow Surgeons: board or committee member; Biomet: paid consultant; Journal of the American Academy of Orthopaedic Surgeons: editorial or governing board, publishing royalties, and financial or material support; Smith & Nephew: IP royalties, paid consultant, and paid presenter or speaker; Springer Nature: publishing royalties and financial or material support; Zimmer: paid consultant and paid presenter or speaker. William N. Levine: Zimmer Biomet Holdings, Inc.: royalties or licenses and consulting fees; American Shoulder and Elbow Surgeons: past president; Journal of American Academy of Orthopaedic Surgeons: editorial board; Journal of Shoulder and Elbow Surgery: editorial board. Any additional authors, their immediate families, and any research foundations with which they are affiliated have not received any financial payments or other benefits from any commercial entity related to the subject of this article.
